# Predictive factors for remission in pediatric Graves’ disease treated with antithyroid drugs: a retrospective study

**DOI:** 10.20945/2359-4292-2026-0089

**Published:** 2026-08-05

**Authors:** Ana João Fernandes, João Pedro Gomes, Rita Aldeia da Silva, Mariana Portela, Sofia Martins, Olinda Marques, Ana Antunes, Maria Miguel Gomes

**Affiliations:** 1 Departamento de Pediatria, Unidade Local de Saúde de Braga, Braga, Portugal; 2 Escola de Medicina, Universidade do Minho, Braga, Portugal; 3 Departamento de Endocrinologia, Unidade Local de Saúde de Braga, Braga, Portugal

**Keywords:** Hyperthyroidism, Graves disease, pediatric endocrinology, antithyroid agents, remission

## Abstract

**Objective:**

Pediatric Graves’ disease (PGD) is a rare autoimmune disorder. Predictors of remission after antithyroid drug (ATD) therapy have been described, but their applicability in pediatric populations remains inconsistent. This study aimed to identify clinical and biochemical factors associated with remission in PGD.

**Subjects and methods:**

Retrospective observational study of children diagnosed with Graves’ disease at a tertiary hospital in Portugal (2001-2021), all initially treated with ATD. Demographic, clinical, and biochemical data at diagnosis, as well as treatment characteristics, were analyzed. The primary outcome was “In Remission”. Secondary outcomes included “Remission Experienced” and “Disease Not Active ≥1 Month”. Comparative analyses, logistic regression, and receiver operating characteristic (ROC) curves were applied.

**Results:**

Among 45,000 pediatric patients, 36 had PGD (prevalence 0.08%). Median age at diagnosis was 13.7 years; 81% were female, and 78% were pubertal. Methimazole was prescribed in 81%. Mean initial treatment duration (ITD) was 35 months, and 47% of patients underwent definitive therapy. At data collection, 18% of patients were in remission. Longer ITD (>24 months) was associated with remission; the optimal cutoff ITD was 51 months. Smaller thyroid volume and TRAb levels at diagnosis correlated with “Disease Not Active ≥1 Month”. Thyroid volume ≥2.5-fold the upper limit of normal (ULN) and TRAb ≥7.055-fold the ULN reduced the likelihood of disease inactivity.

**Conclusion:**

Prolonged ATD therapy, smaller thyroid volume, and lower TRAb levels at diagnosis were associated with favorable outcomes in PGD. The optimal ITD (≥51 months) supports extended ATD therapy. Larger multicenter studies are warranted to confirm these results.

## INTRODUCTION

Pediatric hyperthyroidism is rare, with an estimated incidence of 0.9 per 100,000 in children under the age of 15 years and a clear female predominance. Graves’ disease (GD) accounts for over 95% of cases (^1^-^4^). Its pathogenesis involves genetic, environmental, and immune factors mediated by thyrotropin receptor antibodies (TRAb) that stimulate thyroid hormone overproduction and may coexist with other autoimmune conditions, such as type 1 diabetes or celiac disease (^2^,^3^,^5^). A positive family history in a first-degree relative is observed in ~15% of cases, and the risk of the disease is higher in individuals with Down syndrome or under immunomodulatory therapy (^2^,^3^).

Clinically, pediatric GD (PGD) presents with typical symptoms of hyperthyroidism, *i.e.*, tachycardia, palpitations, hyperphagia, tremors, diarrhea, heat intolerance, anxiety, and insomnia. Over 50% of patients have diffuse goiter, and mild ophthalmopathy may occur. Growth velocity and bone maturation can also be accelerated (^1^-^3^). The diagnosis relies on suppressed thyroid-stimulating hormone (TSH) levels, elevated free triiodothyronine (FT3) and free thyroxine (FT4) levels, and positive TRAb, which are highly specific for GD (^1^,^2^). Thyroid ultrasound, though not required for diagnosis, can aid prognostic assessment, while thyroid scintigraphy is not routinely indicated (^1^,^2^). Antithyroid drugs (ATDs) remain first-line therapy, with radioiodine (^131^I) and thyroidectomy as second-line options (^1^,^3^,^6^,^7^). In children, however, the optimal therapeutic approach remains controversial (^1^,^4^-^10^).

Thionamides, such as methimazole, carbimazole, and propylthiouracil (PTU), inhibit thyroid hormone synthesis, but PTU is avoided in children due to hepatotoxicity (^2^,^3^,^10^). After 2 years of ATD therapy, remission rates are low (20%-30%), though they increase with longer treatment duration, reaching rates of 19.6%, 34.1%, 43.5%, and 50.6% after 3, 5, 7, and 10 years, respectively (^1^,^3^,^4^,^11^,^12^).

Since 2016, the American Thyroid Association (ATA) has acknowledged the potential benefit of extending ATD therapy for GD beyond 2 years (^7^). In contrast, the 2022 European Thyroid Association (ETA) guideline recommends an initial treatment duration (ITD) of at least 3 years (^3^). However, prolonged ATD therapy poses challenges, including the occurrence of adverse effects, requirement for frequent thyroid function monitoring, and poor adherence, especially among adolescents (^4^,^5^,^10^,^13^).

Adverse effects of ATDs, although generally uncommon, must be considered when evaluating prolonged therapy. Minor reactions such as rash, pruritus, or mild gastrointestinal intolerance occur in approximately 5%-25% of pediatric patients, while severe complications like agranulocytosis or hepatotoxicity are rare, with estimated incidences of 0.3%-0.7%. Definitive treatments remain controversial in children due to the potential oncogenic risk of ^131^I, and higher surgical complication rates than in adults (^1^-^4^). These considerations highlight the importance of balancing the risks and benefits of prolonged medical therapy versus early definitive treatment, and of identifying reliable clinical and biochemical predictors of response to ATD that can guide individualized management in PGD (^10^,^14^).

While international studies have explored age, sex, TRAb levels, thyroid volume, and treatment duration as prognostic factors in PGD, results have been inconsistent (^1^,^4^,^6^,^8^-^16^). In Portugal, research is scarce, with only one multicenter study published to date (^6^). Further investigation is needed to clarify prognostic markers and align national practice with international evidence.

In this context, the present study aimed to evaluate clinical and biochemical factors associated with remission in PGD following ATD therapy in patients diagnosed and followed at a Portuguese tertiary hospital between 2001 and 2021.

## SUBJECTS AND METHODS

The study protocol was approved by the Ethics Committee and the Data Protection Office of Braga Hospital. Ethical guidelines and good clinical practice standards were followed, and data confidentiality was maintained throughout the study period.

This retrospective, observational, analytical, longitudinal study included children and adolescents diagnosed with GD at Braga Hospital, a Portuguese tertiary care center, between January 2001 and December 2021. All patients received initial ATD treatment and were followed at the Pediatrics Department and, when applicable, the Pediatric Endocrinology Unit.

A non-random convenience sample was selected. The inclusion criteria were a first consultation within the study period, a GD diagnosis with ATD as the initial therapy, and a follow-up ≥ 6 months. The exclusion criteria included neonatal GD, age > 18 years at diagnosis, and loss to follow-up.

A GD diagnosis required suppressed serum TSH levels, elevated FT3 and/or FT4 levels, and positive TRAb. In some cases, FT3 measurement was unavailable at diagnosis; in these instances, the diagnosis was based on TSH and FT4 levels, along with TRAb results. Consequently, FT3 was excluded from statistical analyses due to missing data.

Treatment followed a dose-titration regimen aiming for euthyroidism (thyroid hormones within reference ranges, followed by TSH). ATD withdrawal was considered after ≥ 24 months of stable euthyroidism on minimal doses (as low as 2.5 mg of methimazole on alternate days) and TRAb levels near or below the upper limit of normal (ULN). Remission was defined as ≥ 12 months of euthyroidism after ATD discontinuation, and relapse as recurrent biochemical hyperthyroidism with positive TRAb after remission.

Throughout the 20-year study period, patients were managed exclusively using a dose-titration regimen. A block-and-replace approach was not employed at any time. Despite changes in ATD preference over time - namely, a shift toward reduced PTU use and increased methimazole use - and modifications to laboratory assay methodologies, clinical decision-making regarding treatment adjustment and ATD withdrawal followed consistent criteria.

Body mass index (BMI) - calculated as weight divided by the square of height (kg/m^2^) and expressed as a standard deviation (SD) score (SDS) by age and sex - was classified as underweight, normal weight, overweight, or obese (^17^,^18^). According to the World Health Organization (WHO) growth reference, for children under 5 years of age, underweight was defined as a BMI-for-age < -2 SD, normal weight as -2 ≤ SD ≤ +2, overweight as +2 < SD ≤ +3, and obesity as SD > +3. For children aged 5 years or older, underweight was defined as BMI-for-age < -2 SD, normal weight as -2 ≤ SD ≤ +1, overweight as +1 < SD ≤ +2, and obesity as SD > +2. Pubertal status was assessed at diagnosis, based on Tanner staging documented at the first endocrinology evaluation, classifying patients as prepubertal or pubertal.

Puberty was defined as Tanner stage ≥ 2 for breast development in girls and Tanner stage ≥ 2 for genital development (testicular volume ≥ 4 mL) in boys (^19^). Thyroid volume was determined by ultrasound using the ellipsoid formula (width x length x depth x 0.52), standardized by the age-specific ULN, through the ratio of measured volume to ULN (7 years: 4.4; 8 years: 4.5; 9 years: 4.9; 10 years: 5.5; 11 years: 6.4; 12 years: 6.7; 13 years: 7.7; 14 years: 7.8). For children younger than 7 years and older than 14 years, ULN values of 4.4 and 7.8, respectively, were applied (^20^,^21^). Not all patients underwent thyroid ultrasound.

Serum FT4 and FT3 levels were measured using competitive chemiluminescent immunoassays, while TSH levels were measured by immunochemiluminescent assay (Atellica IM Analyzer, Siemens Medical Solutions Diagnostics, Erlangen, Germany) and TRAb by radioimmunoassay until 2017 and by chemiluminescent immunoassay from 2017 onwards (IMMULITE 2000 Systems Analyzers, Siemens Medical Solutions Diagnostics). Results were standardized by dividing each by its respective ULN. Reference values for FT4 were 0.9-2.3 ng/mL for ages 0-12 months, 0.8-1.8 ng/mL for ages 1-5 years, 1.0-2.1 ng/mL for ages 6-10 years, and 0.8-1.9 ng/mL for ages 11-18 years. Reference values for TRAb were < 1 U/L or < 9 U/L before 2017 and < 0.55 U/L thereafter (^22^).

**Figure 1:**
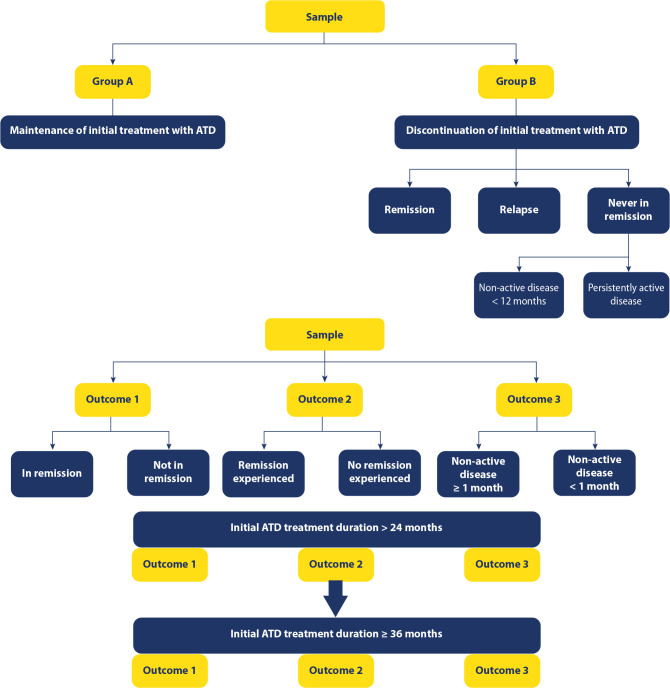
Characterization of the sample according to maintenance or discontinuation of the initial treatment, by Outcomes 1, 2, and 3, and according to the duration of the initial treatment. **Group A:** initial antithyroid drug treatment ongoing at data collection. **Group B:** discontinuation of initial treatment with antithyroid drugs. “Remission”: patients in remission at the time of data collection. “Relapse”: patients who experienced relapse after remission (without achieving a second remission). “Never in Remission”: patients with inactive disease for less than 12 months or with persistently active disease. **Outcome 1:** “In Remission” included patients who were in remission at the time of data collection; “Not in Remission” included all remaining patients, including those who had never discontinued initial antithyroid drug treatment **(Group A). Outcome 2:** “Remission Experienced” grouped patients who had either achieved remission or relapsed after remission (without reaching a new remission). **Outcome 3:** “Disease Not Active ≥ 1 Month” included the sum of patients from the “Remission Experienced” group and those who had inactive disease lasting at least 1 month and less than 12 months.

Data were obtained from medical records. Collected variables included sex, the presence of other autoimmune diseases (AIDs), age at diagnosis, pubertal stage, BMI, thyroid volume, FT4 and TRAb levels, type and dose of initial ATD therapy, and outcome after the initial ATD treatment. The sample was subsequently divided into groups according to the classification schemes shown in **Figure 1**. Group A comprised patients who were still receiving their initial ATD regimen at the time of data collection, while Group B comprised patients who had already discontinued their initial ATD regimen before data collection. Within Group B, patients were further categorized into the following groups: “Remission” (euthyroidism maintained for ≥ 12 months after ATD discontinuation), “Relapse” (suppressed serum TSH levels and elevated FT3 and/or FT4 levels, along with positive TRAb, after achieving remission), and “Never in Remission” (disease inactive for < 12 months or persistently active). For analytical purposes, three outcomes were defined: Outcome 1 - “In Remission”, which included patients in remission at the time of data collection, and “Not in Remission”, which encompassed all remaining patients, including those who had never discontinued initial ATD therapy (Group A); Outcome 2 - “Remission Experienced”, grouping patients who achieved remission at any time, and “No Remission Experienced”, grouping those who never achieved remission or relapsed without attaining a subsequent remission; Outcome 3 - “Disease Not Active ≥ 1 Month”, which included all patients from the “Remission Experienced” group plus those with inactive disease lasting ≥ 1 month but < 12 months after discontinuation of the initial ATD regimen, and those with “Disease Not Active < 1 month”. The selected outcomes were defined to reflect clinically meaningful stages of PGD management. While sustained remission represents the primary therapeutic goal and was therefore defined as the primary outcome (“In Remission”), shorter periods of disease inactivity and remission experienced were included as secondary outcomes, as they are also clinically relevant and frequently inform decisions regarding ATD withdrawal, treatment continuation, and timing of definitive therapy.

ATD: antithyroid drugs.

### Statistical analysis

Statistical analyses were performed using IBM SPSS Statistics, version 29.0 (IBM Corp., Armonk, NY, USA). Statistical significance was defined as p < 0.05, and 95% confidence intervals (CIs) were used to support the interpretation of the results.

The minimum sample size considered representative of the Portuguese pediatric population was n ≥ 16, calculated using an online sample size calculator (^23^), assuming a 95% CI and a 5% margin of error.

Normality of quantitative variables was assessed using the Shapiro-Wilk test, histogram inspection, and evaluation of skewness and kurtosis (^24^). Depending on the normality assumption, parametric or nonparametric tests were applied.

Normally distributed quantitative variables were summarized as mean and SD, while non-normally distributed variables were reported as median and interquartile range. Qualitative variables were expressed as absolute frequencies (n) and/or relative frequencies (%) (^24^).

Comparisons between groups were performed using statistical tests selected according to group dependence (paired or independent), variable type (quantitative or qualitative), and distribution pattern of quantitative variables, with parametric or nonparametric methods applied as appropriate (^25^-^27^).

Binary logistic regression (LR) models - univariate and multivariate - were used to identify potential predictors of outcomes (^28^). Variable selection considered statistical significance in group comparisons, sample size, and the number of variables analyzed. To reduce overfitting, the “1:10 rule” (one predictor per ten positive events) (^29^) was applied, although not strictly, for exploratory purposes, given conflicting evidence in the literature (^30^,^31^).

To minimize multicollinearity and improve robustness, only variables with p < 0.20 in comparative analyses were included in the univariate LR. Those with p < 0.20 in univariate LR were subsequently entered into multivariate LR (^10^). Results were reported as odds ratios (ORs) with 95% CIs. For significant predictors (p < 0.05), Nagelkerke R^2^ was calculated to estimate the variance explained by the model (^28^).

Receiver operating characteristic (ROC) curve analysis was performed for continuous variables of potential clinical interest to assess discriminative ability. For variables with an area under the curve (AUC) > 0.5 - indicating discrimination above chance and improving as AUC approached 1 - the optimal cutoff was determined using the Youden Index (J = Sensitivity + Specificity - 1). A p value < 0.05 indicated statistically significant discriminative capacity (^26^,^28^,^32^).

## RESULTS

**Figure 2:**
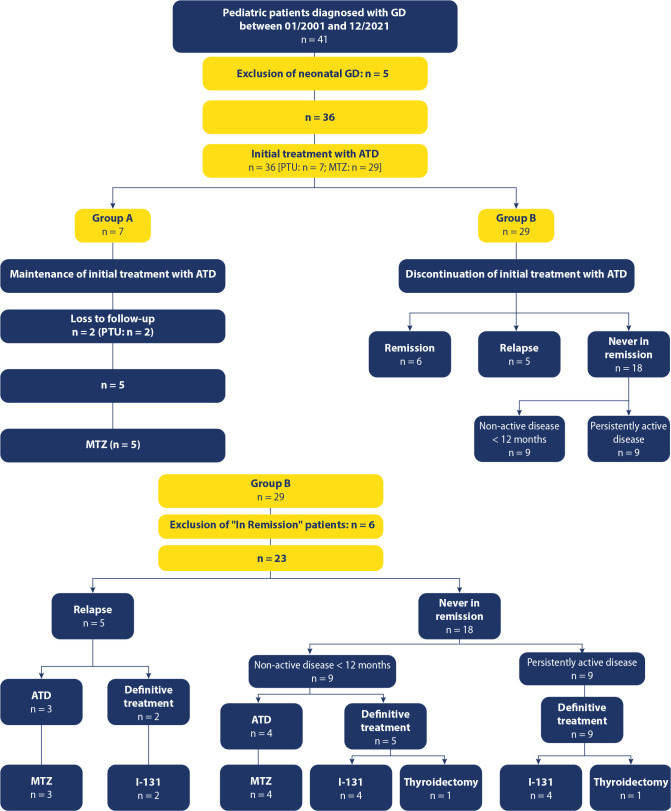
Selection of patients and disease progression according to initial treatment with antithyroid drugs; disease progression in patients who discontinued initial antithyroid drug therapy (Group B) and therapeutic choices made in cases of relapse or lack of remission (antithyroid drugs or definitive therapy with radioiodine or thyroidectomy). **Group A:** maintenance of initial treatment with antithyroid drugs. **Group B:** discontinuation of initial treatment with antithyroid drugs. “Remission”: patients in remission at the time of data collection. “Relapse”: patients who experienced relapse after remission (without achieving a second remission). “Never in Remission”: patients with inactive disease for less than 12 months or with persistently active disease.

ATD: antithyroid drug; GD: Graves’ disease; ^131^I: radioiodine; MTZ: methimazole; PTU: propylthiouracil.

A total of 45,000 children and adolescents were followed at the Pediatrics Department of Braga Hospital from January 2001 to December 2021, of whom 36 were diagnosed with PGD, corresponding to a prevalence of 0.08%. Two patients treated with PTU were subsequently excluded due to loss to follow-up, resulting in a final sample of 34 patients included in the analysis (**Figure 2**). Clinical and biochemical characteristics at diagnosis, along with details of initial ATD treatment and outcomes, are summarized in **Table 1**. Not all patients had complete laboratory or thyroid ultrasound data available due to missing information in the clinical records.

**Table 1:** Descriptive analysis of clinical and biochemical characteristics at diagnosis, details of initial antithyroid treatment, and outcomes

Sex, n (%) Female Male	29 (80.56) 7 (19.44)
Other autoimmune diseases^*^, n (%) Yes	7 (19.44)
Pubertal stage, n (%) Pubertal	28 (77.78)
Body mass index, n (%)† Underweight Normal weight Overweight Obese	1 (2.86) 27 (77.14) 7 (20.00) 0 (0.00)
Initial treatment with antithyroid drugs, n (%) Propylthiouracil Methimazole	7 (19.44) 29 (80.56)
Age (years), median (IQR)	13.67 (4.71)
Standardized thyroid gland volume (xN), median (IQR) (n = 31)^‡^	3.22 (1.94)
Standardized FT4 (xN), median (IQR) (n = 32)^§^	2.15 (1.24)
Standardized TRAb (xN), median (IQR) (n = 29)^¶^	7.11 (14.90)
Initial ATD dose (mg/kg/day), mean ± SD PTU (n=7) Methimazole (n=29)	4.50 ± 1.42 0.36 ± 0.02
Duration of initial ATD treatment (months), median (IQR) (n = 35) PTU, mean ± SD (n = 6)^**^ Methimazole, mean ± SD (n = 29)	31.00 (23.00) 45.50 ± 14.45 33.24 ± 2.48
Duration of initial ATD treatment > 24 months (n = 24) Mean ± SD Median (IQR)	44 ± 3 40 (^19^)
Duration of initial ATD treatment ≥ 36 months (n = 16) Mean ± SD Median (IQR)	51 ± 4 46 (^12^)
Duration of initial ATD treatment until definitive treatment^††^ (n = 16) Mean ± SD Median (IQR)	34 ± 3 40 (^22^)
Duration of initial ATD treatment until I-131 ablation^††^ (n = 13) Mean ± SD Median (IQR)	33 ± 4 39 (^25^)
Duration of initial ATD treatment until thyroidectomy^††^ (n = 3) Mean ± SD Median (IQR)	35 ± 7 40 (.)
Outcomes, n (%) In Remission Remission Experienced Disease Not Active < 12 Months Disease Not Active ≥ 1 Month Relapse	6 (17.65) 11 (32.35) 9 (26.47) 20 (58.82) 5 (45.45)

Results are expressed as mean ± standard deviation, median (interquartile range), or number (%). The n value may vary due to missing data. Standardization: values divided by their respective upper limit of normal.

* Includes type 1 diabetes mellitus (n = 3), celiac disease (n = 3), juvenile idiopathic arthritis (n = 1);

† n = 35 due to 1 missing result in the clinical records;

‡ n = 31, due to 5 missing results in the clinical records;

§ n = 32, due to 4 missing results in the clinical records;

¶ n = 29, due to 7 missing results in the clinical records;

** n = 6, due to 1 patient having a follow-up duration < 6 months;

†† Duration of initial antithyroid drug treatment, in months, for patients who underwent definitive therapy (radioiodine ablation or thyroidectomy). “In Remission” - patients in remission at the time of data collection; “Remission Experienced” - group of patients who achieved remission at any point (patients in remission at data collection plus patients who relapsed and did not achieve a subsequent remission); “Disease Not Active < 12 Months” - group of patients who had a period of inactive disease lasting < 12 months, without achieving remission; “Disease Not Active ≥ 1 Month” - total of patients who experienced remission at any point and those without signs of active disease for at least 1 month but less than 12 months; “Relapse” - patients who experienced remission at any point (n = 11) and subsequently relapsed (n = 5).

FT4: free thyroxine; IQR: interquartile range; SD: standard deviation; TRAb: thyrotropin receptor antibodies.

None of the patients was initially treated with definitive therapy. One patient, who developed PTU-induced toxic hepatitis, discontinued ATD after 4 months and underwent subsequent ^131^I ablation.

In comparative analyses, longer ITD was significantly associated with remission outcomes. Among patients treated for more than 24 months, those “In Remission” had a mean ITD of 58 months compared to 41 months among those “Not in Remission” (p = 0.049). A similar trend was observed for “Remission Experienced” among patients treated for at least 36 months, where it was associated with longer ITD (p = 0.002) (**Table 2**).

**Table 2:** Variables with *p* < 0.20 in the comparative analyses for Outcome 1 (“In Remission” versus “Not in Remission”), Outcome 2 (“Remission Experienced” versus “No Remission Experienced”), and Outcome 3 (“Disease Not Active ≥ 1 Month” versus “Disease Not Active < 1 Month”), as well as for the comparisons on initial treatment durations of more than 24 months and at least 36 months

			Test statistic	*p*
Outcome 1 (n = 34)	In remission (n = 6)	Not in remission (n = 28)		
Standardized thyroid gland volume Mean ± SD (n = 29)	2.54 ± 0.78 (n = 5)	3.57 ± 1.50 (n = 24)	*t*(^27^) = -1.475	0.152
Duration of initial ATD treatment Median (IQR) (n = 34)	48.50 (43.00) (n = 6)	31.00 (19.00) (n = 28)	*U* = 51.500	0.145
Outcome 2 (n = 34)	Remission experienced (n = 11)	No Remission experienced (n = 23)		
Age Median (IQR) (n = 34)	14.92 (4.92) (n = 11)	13.00 (4.16) (n = 23)	*U* = 89.500	0.176
Standardized thyroid gland volume Mean ± SD (n = 29)	2.80 ± 1.20 (n = 8)	3.61 ± 1.49 (n = 21)	*t*(^27^) = -1.367	0.183
Outcome 3 (n = 34)	Disease not active ≥1 month (n = 20)	Disease not active <1 month (n = 14)		
Standardized thyroid gland volume Mean ± SD (n = 29)	2.89 ± 1.14 (n = 17)	4.10 ± 1.58 (n = 12)	*t*(^27^) = 2.400	**0.024**
Standardized TRAb Median (IQR) (n = 27)	6.46 (8.20) (n = 15)	13.84 (51.28) (n = 12)	*U*=133.00	**0.037**
Initial ATD treatment duration > 24 months				
Outcome 1 (n = 24)	In remission (n = 4)	Not in remission (n = 20)		
Standardized thyroid gland volume Mean ± SD (n = 19)	2.62 ± 1.09 (n = 3)	4.06 ± 1.48 (n = 16)	*t*(^17^) = -1.595	0.129
Duration of initial ATD treatment Mean ± SD (n = 24)	58.25 ± 12.61 (n = 4)	40.80 ± 15.70 (n = 20)	*t*(^22^) = 2.080	**0.049**
Outcome 2 (n = 24)	Remission experienced (n=7)	No remission experienced (n = 17)		
BMI SD, n (%) (n = 23) Underweight (n = 1) Normal (n = 18) Overweight (n = 4)	(n = 0) 33.3 (n = 6) 0.0 (n = 0)	100 (n = 1) 66.7 (n = 12) 100 (n = 4)	LR(^2^) =3.448	0.175
Duration of initial ATD treatment Median (IQR) (n=24)	58.43 ± 22.86 (n = 7)	37.65 ± 7.49 (n=17)	*t*(^7^) = 3.354	0.053
Outcome 3 (n=24)	Disease not active ≥ 1 month (n=13)	Disease not active <1 month (n = 11)		
BMI SD, n (%) (n=23) Underweight (n = 1) Normal (n = 18) Overweight (n = 4)	(n = 0) 61.1 (n = 11) 25.0 (n = 1)	100 (n = 1) 38.9 (n = 7) 75.0 (n = 3)	LR(^2^) = 3.286	0.193
Standardized TRAb Median (IQR) (n = 19)	6.84 (9.60) (n = 10)	15.56 (79.32) (n = 9)	*U* = 71.000	**0.035**
Duration of initial ATD treatment Mean ± SD (n = 24)	48.62 ± 20.42 (n = 13)	37.91 ± 6.99 (n = 11)	*t(*15) = 1.772	0.096
Initial ATD treatment duration ≥ 36 months				
Outcome 1 (n = 16)	In remission (n = 4)	Not in remission (n = 12)		
Standardized thyroid gland volume Mean ± SD (n = 12)	2.62 ± 1.09 (n = 3)	3.62 ± 0.92 (n = 9)	*t*(^10^) = -1.568	0.148
Duration of initial ATD treatment Median (IQR) (n = 16)	57.00 (24.00) (n = 4)	42.50 (10.0) (n = 12)	*U* = 8.500	0.058
Outcome 2 (n = 16)	Remission experienced (n = 6)	No remission experienced (n = 10)		
Standardized thyroid gland volume Mean ± SD (n = 12)	2.62 ± 1.09 (n = 3)	3.62 ± 0.92 (n = 9)	*t*(^10^) = -1.568	0.148
Duration of initial ATD treatment Median (IQR) (n = 16)	57.00 (30.00) (n = 6)	41.00 (8.00) (n = 10)	*U* = 3.000	**0.002**
Outcome 3 (n = 16)	Disease not active ≥ 1 month (n = 10)	Disease not active <1 month (n = 6)		
Standardized TRAb Median (IQR) (n = 11)	6.67 (8.20) (n = 7)	13.84 (76.94) (n = 4)	*U* = 24.000	0.073
Duration of initial ATD treatment Median (IQR) (n = 16)	50.50 (24.0) (n = 10)	41.50 (8.00) (n = 6)	*U* = 16.500	0.147

Results are expressed as mean ± standard deviation, median (interquartile range), or number (%); the n value may vary due to missing data. Standardization: values divided by their respective upper limit of normal. “In Remission”: patients in remission at the time of data collection. “Remission Experienced”: patients who experienced remission at some point (those in remission at the time of data collection, as well as those who relapsed without subsequently achieving a new remission). “Disease Not Active ≥ 1 Month”: patients who experienced remission at some point, as well as those with inactive disease for a period of at least 1 month and less than 12 months.

ATD: antithyroid drug; BMI: body mass index; IQR: interquartile range; SD: standard deviation; TRAb: thyrotropin receptor antibodies.

Lower thyroid volume and TRAb levels at diagnosis correlated with “Disease Not Active ≥1 Month” (p = 0.024 and p = 0.037, respectively). For initial therapy longer than 24 months, standardized TRAb levels remained significantly lower in this group (p = 0.035) (**Table 2**).

Comparing the group of patients that maintained the initial ATD treatment with the group that discontinued it, only standardized TRAb levels were significantly higher: 31.64 (84.10) in Group A versus 6.57 (10.24) in Group B (p = 0.008).

In univariate LR, ITD was a significant predictor of “Remission Experienced” in the group with initial therapy longer than 24 months (p = 0.041; OR = 1.14; 95% CI: 1.005-1.297), explaining 47% of outcome variability. For “Disease Not Active ≥ 1 Month”, thyroid volume was initially significant (p = 0.042; OR = 0.49) but lost significance after adjustment for TRAb levels (**Table 3**). No further univariate or multivariate LR results reached significance.

**Table 3:** Odds ratio of variables selected from the comparative analyses between groups (Table 2) for the occurrence of the events “In Remission”, “Remission Experienced”, and “Disease Not Active ≥ 1 Month”

	Variable	Unadjusted OR				Adjusted OR		
		OR	95% CI	*p*		OR	95% CI	*p*
Outcome 1	Standardized thyroid gland volume	0.462	0.156-1.362	0.162^*^		0.345	0.080-1.490	0.154
Duration of initial ATD treatment	1.033	0.987-1.082	0.162^*^		1.078	0.977-1.191	0.136
Outcome 1 (>24 months)	Standardized thyroid gland volume	0.310	0.064-1.504	0.146^*^		0.063	0.000-8.311	0.267
	Duration of initial ATD treatment	1.058	0.990-1.132	0.099^*^		1.800	0.787-4.117	0.164
Outcome 1 (≥36 months)	Standardized thyroid gland volume	0.311	0.059-1.630	0.167^*^		-	-	-
	Duration of initial ATD treatment	1.038	0.967-1.115	0.297		-	-	-
Outcome 2	Age	1.090	0.869-1.367	0.456		-	-	-
	Standardized thyroid gland volume	0.602	0.283-1.280	0.188^*^		-	-	-
Outcome 2 (> 24 months)	BMI SD	0.376	0.035-4.037	0.420		-	-	-
	Duration of initial ATD treatment	1.142	1.005-1.297	0.041^*^		-	-	-
Outcome 2 (≥36 months)	Standardized thyroid gland volume	0.311	0.059-1.630	0.167^*^		0.064	0.000-8.395	0.269
	Duration of initial ATD treatment	1.483	0.958-2.296	0.077^*^		1.793	0.778-4.129	0.170
Outcome 3	Standardized thyroid gland volume	0.492	0.249-0.975	0.042^*^		0.519	0.217-1.244	0.142
	Standardized TRAb	0.943	0.871-1.020	0.143^*^		0.937	0.846-1.038	0.211
Outcome 3 (>24 months)	BMI SD	0.607	0.094-3.943	0.601		-	-	-
	Standardized TRAb	0.923	0.818-1.041	0.190^*^		0.923	0.811-1.053	0.226
	Duration of initial ATD treatment	1.060	0.981-1.146	0.141^*^		1.053	0.933-1.188	0.403
Outcome 3 (≥36 months)	Standardized TRAb	0.902	0.718-1.132	0.373		-	-	-
	Duration of initial ATD treatment	1.143	0.936-1.395	0.190^*^		-	-	-

Variables with *p* < 0.20 in the comparative analysis between groups were included in the univariate logistic regression (unadjusted odds ratio [OR]). Those with *p* < 0.20 in the univariate logistic regression (^*^) were included in the multivariate logistic regression (adjusted OR). Adjusted ORs with a *p* < 0.05 were considered statistically significant. Standardization: values divided by their respective upper limit of normal. “In Remission”: patients in remission at the time of data collection. “Remission Experienced”: patients who experienced remission at some point (patients in remission at the time of data collection, as well as those who relapsed without subsequently achieving a new remission). “Disease Not Active ≥ 1 Month”: patients who experienced remission at some point, as well as those with inactive disease for a period of at least 1 month and less than 12 months.

ATD: antithyroid drug; BMI: body mass index; OR: odds ratio; SD: standard deviation; TRAb: thyrotropin receptor antibodies.

**Figure 3:**
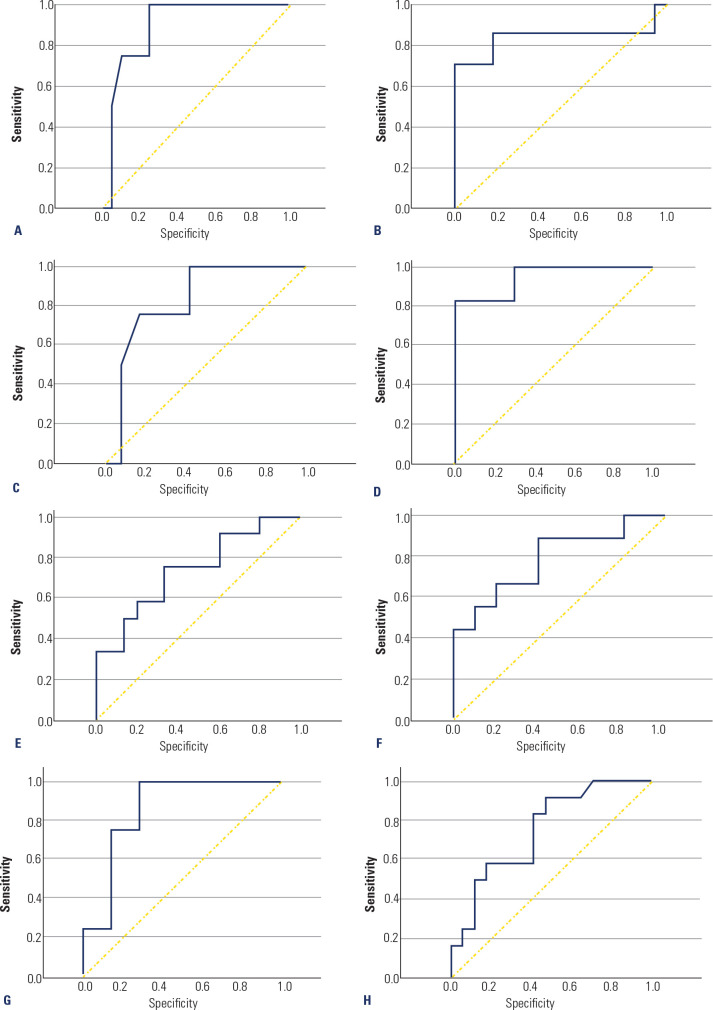
Receiver operating characteristic (ROC) curves. (**A**) ROC curve assessing the discriminative ability of the variable “Initial Treatment Duration” for the event “In Remission” (**Outcome 1**, treatment > 24 months). (**B**) ROC curve assessing the discriminative ability of the variable “Initial Treatment Duration” for the event “Remission Experienced” (**Outcome 2**, treatment > 24 months). (**C**) ROC curve assessing the discriminative ability of the variable “Initial Treatment Duration” for the event “In Remission” (**Outcome 1**, treatment ≥ 36 months). (**D**) ROC curve assessing the discriminative ability of the variable “Initial Treatment Duration” for the event “Remission Experienced” (**Outcome 2**, treatment ≥ 36 months). (**E**) ROC curve assessing the discriminative ability of the variable “Standardized TRAb” for the event “Disease Not Active ≥ 1 Month” (**Outcome 3**).

An analysis of the ROC curve confirmed the discriminative ability of ITD for remission outcomes. For therapy longer than 24 months, ITD showed excellent performance (AUC = 0.894 for “In Remission” and 0.840 for “Remission Experienced”), with an optimal cutoff of 51 months. For treatment ≥ 36 months, the discriminative ability remained high (AUC = 0.823 and 0.950, respectively) (**Figure 3**).

Standardized TRAb levels demonstrated acceptable discrimination for “Disease Not Active ≥ 1 Month” (AUC = 0.739, p = 0.036), improving to excellent for treatment ≥ 36 months (AUC = 0.857, p = 0.059). The optimal TRAb cutoff point was 7.055-fold the ULN (**Figure 3**).

For standardized thyroid volume, discrimination was acceptable for “Disease Not Active ≥ 1 Month” (AUC = 0.752, p = 0.023), with an optimal cutoff point of 2.50 (**Figure 3**).

## DISCUSSION

The optimal treatment for GD in children remains controversial due to the limited efficacy of available therapeutic options and the difficulty in predicting remission. Although ATDs are the recommended first-line therapy (^3^,^7^,^9^), long-term remission after 2 years is achieved in only 20%-30% of patients (^1^,^3^,^13^). The ETA currently advises at least 3 years of ATD treatment, extendable to 5 or more years depending on clinical and biochemical evolution (^3^). Identifying reliable prognostic markers at diagnosis could help individualize therapy and avoid unnecessary exposure to prolonged medication or premature definitive treatment.

Baseline characteristics in our sample - predominance of females, pubertal stage at diagnosis, and normal BMI - were consistent with previous studies (^4^-^6^,^10^,^13^,^14^). Notably, PTU was prescribed only before 2010, reflecting historical practice. Following INFARMED’s (National Authority of Medicines and Health Products, I.P. in Portugal) 2010 recommendation highlighting the hepatotoxicity risk of PTU, its use in pediatric patients was gradually discontinued, and methimazole became the preferred first-line ATD in accordance with national and international guidelines.

Longer ITD was associated with remission outcomes, suggesting a potential benefit of extended therapy. Analysis of the ROC curve identified an optimal ITD of approximately 51 months, consistent with ETA recommendations and other reports showing increased remission rates with prolonged therapy (^1^,^3^,^4^). Studies by Léger and cols. and Ohye and cols. also observed higher remission rates after 4-6 years of ATD treatment (^11^,^12^). These findings support the rationale for maintaining ATD therapy for at least 3 years.

Smaller thyroid volume at diagnosis was associated with disease inactivity, in line with previous studies reporting poorer outcomes in patients with larger goiters (^9^). Analysis of the ROC curve in our sample suggested that a thyroid volume ≥ 2.5-fold the ULN reduced the likelihood of disease inactivity.

Lower TRAb levels at diagnosis were also associated with better outcomes. Similar trends have been reported by Gastaldi and cols. (^8^) and Rho and cols. (^14^), who showed that persistently high TRAb titers predicted relapse or failure to achieve remission. Our cutoff of 7.0-fold the ULN aligns with these results and supports the role of TRAb monitoring in guiding clinical decisions.

No statistically significant associations were found between remission outcomes and sex, pubertal stage, BMI, FT4 levels, or the type of initial ATD used (PTU versus methimazole), consistent with prior reports (^8^,^14^,^33^). Although the coexistence of other AIDs was more frequent among patients who experienced remission or relapse, this trend did not reach statistical significance and warrants further investigation. A similar observation was reported by Gatta and cols., who suggested that concurrent autoimmune conditions might modulate immune activity and favor GD remission. According to the GRAPHE study, the most reasonable hypothesis is that the immunological cascade involving multiple autoimmune diseases may contribute to shifts in TRAb profiles, particularly from stimulating to blocking antibodies, together with a lower overall immune response potentially related to differences in B-cell subsets (^34^).

Although our findings support the potential benefit of prolonged ATD therapy, the clinical implications of extended treatment must be interpreted with caution. This study did not systematically evaluate treatment-related adverse effects or adherence to long-term oral therapy, both of which are clinically relevant considerations, particularly in pediatric and adolescent populations. While serious adverse events associated with ATDs are uncommon, prolonged treatment may increase the cumulative burden of minor side effects and pose challenges to sustained adherence, potentially influencing real-world effectiveness. Therefore, decisions regarding extended ATD therapy should be individualized, balancing the potential benefits of remission against treatment tolerability, patient adherence, and family preferences.

Importantly, although several variables demonstrated significant associations with remission-related outcomes in univariate analyses, no independent predictors remained statistically significant after multivariate adjustment. This likely reflects limited statistical power due to the small sample size and the low number of outcome events. Therefore, the identified associations should be interpreted as exploratory and hypothesis-generating, rather than establishing independent predictive or causal relationships. Larger, adequately powered multicenter studies are required to confirm these findings and to determine whether these factors retain independent prognostic value in PGD.

### Limitations

This study has several limitations. The small sample size and retrospective single-center design limited statistical power, particularly for subgroup analyses, and did not allow standardized long-term follow-up. Missing clinical, laboratory, and imaging data further constrained the analyses. Inter-laboratory variability and suboptimal standardization methods over the long study period may have influenced biochemical measurements. In addition, the small number of outcome events reduces the reliability of multivariate models and ROC curve analyses; therefore, any derived cutoff points or predictive estimates should be considered exploratory rather than confirmatory. Larger prospective multicenter studies with standardized follow-up are needed to validate these findings and refine prognostic markers in PGD.

In conclusion, in this 20-year retrospective cohort study, prolonged ATD therapy was associated with higher remission rates, supporting treatment durations of at least 3 years and identifying an optimal cutoff of around 51 months. In addition, smaller thyroid volume and lower TRAb levels at diagnosis were associated with disease inactivity, suggesting a supportive role of these markers in clinical risk stratification. Given the small sample size and the retrospective single-center design, further multicenter studies are needed to validate these associations and refine prognostic tools for clinical practice.

## Data Availability

datasets related to this article will be avail-able upon request to the corresponding author.
